# Decreased Expression of Nuclear p300 Is Associated with Disease Progression and Worse Prognosis of Melanoma Patients

**DOI:** 10.1371/journal.pone.0075405

**Published:** 2013-09-30

**Authors:** Anand Rotte, Madhuri Bhandaru, Yabin Cheng, Cecilia Sjoestroem, Magdalena Martinka, Gang Li

**Affiliations:** 1 Department of Dermatology and Skin Science, University of British Columbia, Vancouver, British Columbia, Canada; 2 Department of Pathology & Laboratory Medicine, University of British Columbia, Vancouver, British Columbia, Canada; University of Tennessee, United States of America

## Abstract

**Background:**

Genomic instability due to UV radiation is one of the leading causes for melanoma. Histone acetyltransferase p300 plays an indispensible role in DNA repair and maintenance of genomic integrity. The present study was performed to analyze the correlation between p300 expression, melanoma progression and patient survival.

**Methods:**

Tissue microarray and immunohistochemical analysis was employed to study the expression of p300 in melanoma patients. A total of 358 melanoma patients (250 primary melanoma and 108 metastatic melanoma) were used for the study. Kaplan-Meier, univariate and multivariate Cox regression analysis, and receiver-operating characteristic curves, were used to elucidate the prognostic significance of p300 expression.

**Results:**

Our results demonstrate that p300 is expressed in both nucleus and cytoplasm but the nuclear expression of p300 is predominant. The progression of disease from dysplastic nevi to primary melanoma and to metastatic melanoma was associated with decreased nuclear and increased cytoplasmic p300 expression. Especially, the loss of nuclear and gain in cytoplasmic p300 was correlated with the progression of melanoma from AJCC stage II to stage III, which requires the migration and metastasis of cancer cells from primary sites to lymph nodes. Similarly, decrease in nuclear, and increase in cytoplasmic p300 expression correlated with worse survival of melanoma patients. Nuclear p300 but not cytoplasmic p300 could predict the patient survival independent of AJCC stage, age and gender.

**Conclusion:**

Loss of nuclear p300 expression is an indicator of worse patient survival and is an independent prognostic marker for melanoma.

## Introduction

p300, also known as E1A binding protein p300 or EP300, was originally identified in protein interaction assays through its interaction with the adenoviral transforming protein E1A [1]. It is known to regulate a broad range of cellular activities like proliferation, cell cycle regulation, apoptosis, differentiation and DNA damage response [2]. Though p300 is known to act by different mechanisms, acetylation of nuclear histones at lysine residues and causing chromatin relaxation is understood to be the primary mode of action [3]. Relaxation of chromatin allows various repair factors to get access to the sites of DNA lesions which are not normally accessible [4], [5]. Damaged DNA if left unrepaired can have deleterious effects on genomic integrity and can lead to mutations in oncogenes or tumor suppressor genes. Histone acetyltransferases (HATs) thus play an indispensible role in the repair of DNA and maintenance of genomic stability [2], [4]. Along these lines, analysis of tumors corresponding to breast, lung, colon cancers and their normal counterparts, revealed an association between decreased levels of acetylated and trimethylated histone H4 and patient survival [6], [7], [8]. Among all the members of the HAT super family, cyclic AMP response element-binding protein (CBP) and p300 are the only HATs that are reported to be capable of acetylating all the four histones suggesting their importance in the global cellular histone acetylation [2]. Accordingly, inactivating mutations of p300 and CBP have been reported in follicular lymphoma, diffuse large B-cell lymphoma, small cell lung cancer and endometrial cancer [9], [10], [11]. On the other hand, increased expression of p300 has been reported to correlate with cancer progression and patient survival [12], [13]. However, to our knowledge, the exact role of p300 in development and progression of melanoma has never been studied. The present study was therefore undertaken to analyze the correlation between p300 expression and patient survival using the tissue samples collected from melanoma patients.

## Patients and Methods

### Cell Culture

The MM-RU, MM-AN and MM-LH melanoma cell lines were kind gifts from Dr. H. R. Byers [14]. A375 cell line was purchased from the American Type Culture Collection (ATCC). All the melanoma cell lines were maintained in Dulbecco’s Modified Eagle Medium (DMEM) supplemented with 10% fetal bovine serum (Invitrogen, Burlington, ON, Canada) in the presence of 100 units/ml penicillin, 100 µg/ml streptomycin and 25 µg/ml amphotericin B. Normal human melanocytes (NHM), obtained from (ScienCell, Carlsbad, CA, USA) were cultured in Human Melanocyte Medium (ScienCell) supplemented with manufacturer recommended growth factors.

### Immunofluorescence

To visualize the expression of endogenous p300, cells were cultured on cover slips for 24–48 h, depending on the type of experiment and then were fixed with 1% paraformaldehyde in PBS for 10 min at room temperature and permeabilized with 1% Triton-X 100 in PBS for 10 min. The slides were incubated with mouse anti-p300 (Millipore, Billerica, MA, USA) followed by incubation with Alexa Fluor 488 anti-mouse (Invitrogen). The slides were mounted with Vectorshield mounting media with DAPI (Vector Laboratories, Burlington, ON, Canada). The images were obtained by a laser scanning confocal microscope, LSM 780, equipped with the ZEN software, under the 10× eyepiece and 63× oil immersion lens (Carl Zeiss, Toronto, ON, Canada). Ten to fifteen optical sections each with 0.4 µm distance in the z-direction were obtained for each image. The images were further processed into 2D by maximum intensity projection provided from the ZEN software.

### Ethics Statement

The use of human skin tissues and the waiver of patient consent in this study were approved by the Clinical Research Ethics Board of the University of British Columbia [15]. The study was conducted according to the principles expressed in the Declaration of Helsinki.

### Patient Specimens and Tissue Microarray Construction

The collection of patient specimens and the construction of the tissue microarray (TMA) have been previously described [16]. Briefly, we used the patient data collected from 1990 to 1999 (referred to as “training set”) for examining the correlation between p300 expression and patient survival. The findings from the initial training set were further validated by data collected between 2000 and 2009 (referred to as “validation set”). Of the 748 patient specimens collected, 474 biopsies including 392 melanoma (183 cases in the training set and 209 cases in the validation set) and 82 cases of nevi (28 normal nevi and 54 dysplastic nevi) could be evaluated for p300 staining in this study, due to loss of biopsy cores or insufficient tumor cells present in the cores. The criterion for patient exclusion and inclusion and the demographic characteristics of melanoma patients are detailed in Figure S1, and Table S1. There were no significant differences in the distribution of the age, sex and American Joint Committee on Cancer (AJCC) stages between the training and the validation sets. Where needed, the two data sets were subsequently combined to increase the study power and to facilitate the analysis of survival in primary and metastatic melanoma patients separately. All specimens were obtained from the archives of the Department of Pathology, Vancouver General Hospital. The most representative tumor area was carefully selected and marked on the hematoxylin and eosin stained slides and the TMAs were assembled using a tissue-array instrument (Beecher Instruments, Silver Spring, MD). Tissue cores of 0.6-mm thickness were taken in duplicate from each biopsy. Using a Leica microtome, multiple 4 µM sections were cut and transferred to adhesive-coated slides using regular histological procedures. One section from each TMA was routinely stained with hematoxylin and eosin whereas the remaining sections were stored at room temperature for immunohistochemical staining.

### Immunohistochemistry

Tissue microarray (TMA) slides were dewaxed at 55°C for 20 min followed by three 5 min washes with xylene. The tissues were then rehydrated by washing the slides for 5 min each with 100%, 95%, 80% ethanol and finally with distilled water. The slides were then heated to 95°C for 30 min in 10 mmol/L sodium citrate (pH 6.0) for antigen retrieval and then treated with 3% hydrogen peroxide for 1 hour to block the endogenous peroxidase activity. After blocking the slides with the universal blocking serum (Dako Diagnostics, Carpinteria, CA, USA), the sections were incubated overnight with monoclonal mouse anti-p300 antibody (1∶50 dilution; Millipore, USA) at 4°C. The sections were then incubated for 30 min with a biotin-labeled secondary antibody and then with streptavidin-peroxidase (Dako Diagnostics). The samples were developed by treatment with 3,3′-diamino-benzidine substrate (Vector Laboratories, Burlington, Ontario, Canada) and with hematoxylin to counter-stain the nuclei. Negative controls were done by omitting the p300 antibody during the primary antibody incubation.

### Evaluation of Immunostaining

The evaluation of p300 staining was done blindly by microscopic examination of the tissue sections by one dermatopathologist and two other observers simultaneously, using a multiple viewing microscope and a consensus was reached for score of each core. p300 staining intensity was scored as 0+, 1+, 2+, 3+ whereas the percentage of p300 positive cells was scored as 1 (1–25%), 2 (26–50%), 3 (51–75%) and 4 (76–100%). In the cases of discrepancy between duplicated cores, the higher score from the two tissue cores was taken as the final score. The product of intensity and percentage was taken as the immunoreactive score (IRS) [17]. Based on IRS, p300 staining in the tissue sections was categorized as negative (IRS 0), weak (IRS 1–4), moderate (IRS 6–8), and strong (IRS 9–12). Since p300 was found to be expressed in both nucleus and cytoplasm, the staining was evaluated accordingly, and at the same time. The immunoreactive score of nuclear staining was referred to as “N-IRS” and the cytoplasmic staining was referred to as “C-IRS”. The optimum cut-off values for the IRS were derived based on the IRS pattern in nevi and using the Receiver-Operating Characteristic (ROC) and the area under the ROC curves (see below). The cut-off points were additionally confirmed using the X-tile software (Yale University).

### Determination of Cut-off Values Using Receiver-Operating Characteristic (ROC) Curves

Briefly, ROC curves were obtained by plotting the true positive rate (Y-axis) verses the false positive rate (X-axis) for the different possible cut-off points of the diagnostic test [18]. An ROC curve demonstrates both the sensitivity (true positive rate, Y-axis) and the specificity (false positive rate, X-axis) of the particular marker. Assuming that the higher values of the marker indicate progression of the disease, the area under the curve (AUC) and diagnostic accuracy of the marker increases as the curve gets closer towards the left-hand border and the top border of the plot, whereas, as the curve gets closer to the diagonal reference line in the plot, the AUC and accuracy decreases. In the case the lower values of the marker indicate progression of the disease, the diagnostic accuracy of the marker increases as the curve gets closer towards the right-hand border and the lower border of the plot. Area under the ROC curve is thus taken as a measure of diagnostic accuracy for the particular cut-off and for our analysis, the area under the ROC curves were obtained with various possible cut-off values (0, 1, 2, 3, 4, 6, 8, 9). Thus, when the expression level of the marker increased from nevi to melanoma, the cut-off that has largest area under the curve was taken as the cut-off that best describes the difference between nevi and melanoma cores. On the other hand when the expression level decreased, the cut-off that had the smallest AUC was chosen as the best cut-off.

### Statistical Analysis

Correlation between p300 and clinicopathologic parameters was evaluated by Kruskal-Wallis test and Chi-square test among patient subgroups. Survival time was calculated from the date of melanoma diagnosis to the date of death or last follow-up. The effect of p300 expression on the overall and disease-specific survival was evaluated by Kaplan-Meier analysis and log-rank test. Additionally, univariate and multivariate Cox proportional hazards regression models were preformed to estimate the crude hazard ratios (HRs) or adjusted HRs and their 95% confidential intervals (CIs). *P*-value <.05 was considered as statistically significant. All the statistical analyses were performed using SPSS version 16.0 (SPSS Inc, Chicago, IL) software and Graph pad prism 4.0 (Prism, USA) software.

## Results

### p300 Expression in Melanoma

**Figure 1:**
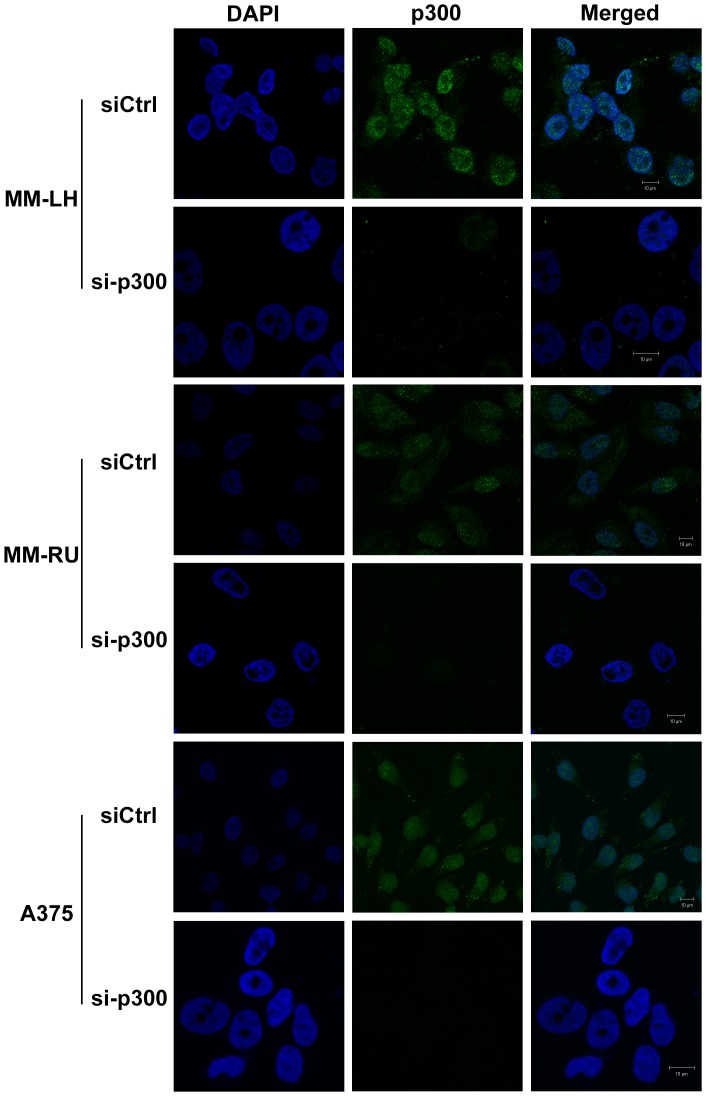
p300 is expressed in both nucleus and cytoplasm Silencing of p300 leads to abrogation of p300 expression in both nucleus and cytoplasm. Cells were grown on cover slips and transfected either with control siRNA (siCtrl) or p300 siRNA (si-p300) and after 48 hrs fixed as described in the methods section. Immunofluorescent staining was performed on the cells grown on cover slips using anti-p300 antibody (green). Nucleus was stained with DAPI (blue).

Confocal microscopy was employed to visualize the p300 expression pattern in cultured melanoma cells. As seen in Figure S2, p300 was expressed in melanocytes and in all the melanoma cell lines tested. Furthermore, the nucleus was the primary site of expression in all the cell lines, but in some cell lines (MM-RU and A375), p300 was also present in the cytoplasm (Figure S2). In order to confirm that p300 was expressed in both nucleus and cytoplasm in MM-RU and A375 cells, we silenced the endogenous p300 using respective siRNA and then stained the cells. As seen in Figure 1, silencing of p300 led to complete abrogation of signals from both nucleus and cytoplasm indicating that p300 was indeed localized in cytoplasm and nucleus.

### Nuclear p300 Expression is Reduced & Cytoplasmic p300 Expression is Increased in Melanoma

**Figure 2:**
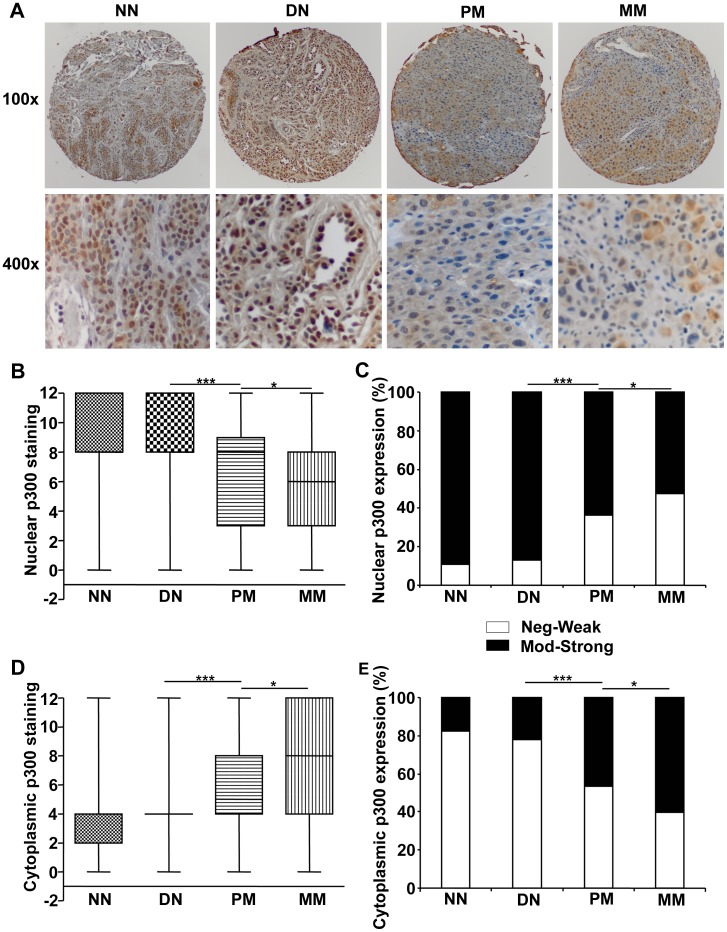
p300 expression in melanoma patients. (A) Representative images at 100× (upper panel) and 400× (lower panel) magnification of normal nevi (NN) and dysplastic nevi (DN) with strong and primary melanoma (PM) and metastatic melanoma (MM) with weak nuclear p300 staining. (B) Nuclear p300 staining is significantly decreased from normal and dysplastic nevi to melanoma by Kruskal-Wallis test. (C) Percentage of strong p300 staining in nuclei is significantly decreased from normal and dysplastic nevi to melanoma by χ^2^ test. (D) Cytoplasmic p300 staining is significantly increased from nevi to melanoma as studied by Kruskal-Wallis test. (E) Percentage of strong cytoplasmic staining is significantly increased from nevi to melanoma by χ^2^ test. **P<.01, ***P<.001.

As seen in the cultured cells, p300 staining was seen both in the nucleus and cytoplasm in our tissue samples from melanoma patients (Figure S3, Figure 2A). IRS scores for the expression of nuclear p300 revealed that there was a subtle increase of p300 expression in nuclei from normal nevi to dysplastic nevi and then a decrease from dysplastic nevi to primary melanoma and further to metastatic melanoma. The percentage of negative cases (N-IRS 0), 7.1% in normal nevi and 3.7% in dysplastic nevi, were increased to 20.7% in primary melanoma and to 20.4% in metastatic melanoma (Figure S4A). On the other hand, the percentage of cases expressing strong nuclear p300 (N-IRS 9 and 12), 39.3% in normal nevi and 46.3% in dysplastic nevi, was decreased to 25.9% in primary melanoma and 20.6% in metastatic melanoma. Based on the area under the ROC curve values, the best cut-off IRS was determined as N-IRS 4 (Table 1) and the analysis of the data was carried out by using the N-IRS 4 as cut-off. The data was then divided into low (negative-to-weak) expression (N-IRS 0, 1, 2, 3 and 4) and high (moderate-to-strong) expression (N-IRS 6, 8, 9 and 12). Accordingly, the percentage of cases with low nuclear p300 staining increased significantly from normal and dysplastic nevi to primary melanoma and to metastatic melanoma (Table 2, Figure 2B, C). But, the trend of increased p300 expression from normal to dysplastic nevi did not reach statistical significance (Table 2).

**Table 1:** Area under the ROC curve values for nuclear and cytoplasmic p300 expression.

IRS cut-off	Nuclear p300	Cytoplasmic p300
0 vs 1 to 12	0.416	0.537
0 & 1 vs 2 to 12	0.412	0.534
0 to 2 vs 3 to 12	0.412	0.547
0 to 3 vs 4 to 12	0.402	0.558
0 to 4 vs 6 to 12	**0.360**	**0.655**
0 to 6 vs 8 to 12	0.363	0.643
0 to 8 vs 9 to 12	0.403	0.568
0 to 9 vs 12	0.407	0.568

**Table 2:** p300 staining and clinicopathological characteristics of 392 melanoma patients.

Variables	Nuclear p300			Cytoplasmic p300		
	low	high	*p*–value*	low	high	*p*-value*
**All Melanoma**						
Age						
≤60	85 (42.5%)	115 (57.5%)	.388	100 (50.0%)	100 (50.0%)	.569
>60	73 (38.2%)	118 (61.8%)		90 (47.1%)	101 (52.9%)	
Gender						
Male	94 (39.8%)	136 (60.2%)	.824	111 (49.1%)	119 (50.9%)	.874
Female	64 (40.9%)	97 (59.1%)		79 (48.3%)	82 (51.7%)	
AJCC stage						
I	41 (32.8%)	84 (67.2%)	.029	81 (64.8%)	44 (35.2%)	.0005
II	51 (40.5%)	75 (59.5%)		61 (48.5%)	65 (51.5%)	
III	33 (55.9%)	26 (44.1%)		20 (33.9%)	39 (66.1%)	
IV	34 (41.5%)	48 (58.5%)		38 (46.3%)	44 (53.7%)	
Site						
Sun Protected	118 (39.5%)	181 (60.5%)	.460	158 (52.8%)	141 (47.2%)	.319
Sun Exposed	41 (44.1%)	52 (55.9%)		42 (45.2%)	51 (55.8%)	
Site (Cases with age >60)						
Sun Protected	54 (38.3%)	87 (68.1%)	.180	67 (47.5%)	74 (52.5%)	.319
Sun Exposed	25 (49.0%)	26 (51.0%)		25 (49.0%)	26 (51.0%)	
**Primary Melanoma**						
Age						
≤60	45 (37.2%)	76 (62.8%)	.802	71 (58.7%)	50 (41.3%)	.118
>60	47 (36.2%)	83 (63.8%)		64 (49.3%)	66 (50.7%)	
Gender						
Male	54 (38.2%)	84 (60.8%)	.408	74 (53.8%)	64 (46.2%)	.913
Female	38 (33.6%)	75 (66.4%)		61 (54.0%)	52 (46.0%)	
Tumor Thickness						
≤2	46 (33.6%)	91 (66.4%)	.306	85 (62.0%)	52 (38.0%)	.003
>2	46 (40.4%)	68 (59.6%)		50 (45.6%)	64 (54.4%)	
Ulceration						
Absent	69 (34.8%)	129 (65.2%)	.251	118 (59.6%)	80 (40.4%)	.093
Present	23 (43.4%)	30 (56.6%)		24 (45.3%)	29 (54.7%)	
Subtype						
Acrolentigous	4 (50.0%)	4 (50.0%)	.556	2 (25.0%)	6 (75.0%)	.603
Lentigous	18 (41.9%)	25 (58.1%)		24 (55.8%)	19 (44.2%)	
Nodular	18 (40.0%)	27 (60.0%)		25 (55.6%)	20 (44.4%)	
Spindle type	4 (40.0%)	6 (60.0%)		7 (70.0%)	3 (30.0%)	
Superficially spreading	25 (28.7%)	62 (71.3%)		50 (57.5%)	37 (42.5%)	
Unspecified	23 (39.7%)	35 (60.3%)		33 (56.9%)	25 (43.1%)	
**Metastatic Melanoma**						
Age						
≤60	39 (49.4%)	40 (50.6%)	.492	29 (36.7%)	50 (63.3%)	.409
>60	27 (43.5%)	35 (56.5%)		27 (43.5%)	35 (56.5%)	
Gender						
Male	41 (44.1%)	52 (55.9%)	.843	38 (40.9%)	55 (59.1%)	.798
Female	22 (45.8%)	26 (54.2%)		18 (37.5%)	30 (62.5%)	

Sun-protected sites: trunk, arm, leg and feet; Sun-exposed sites: head and neck.

* χ^2^ test.

In contrast to nuclear expression, p300 expression in the cytoplasm was increased in melanoma cases compared to nevi cases. The percentage of negative cases (C-IRS 0) decreased from 21.4% in normal nevi to 14.8% in dysplastic nevi and to 8.4% and 9.2% in primary and metastatic melanoma respectively (Figure S4B). The percentage of cases expressing strong (C-IRS 9 and 12) cytoplasmic p300 was slightly decreased in dysplastic nevi (3.7%) as compared to normal nevi (7.1%). However, the expression was increased to 15.2% in primary melanoma and to 23.4% in metastatic melanoma (Figure S4B). As described above, the best cut-off was determined as C-IRS 4 and the data were divided into low (negative-to-weak) (C-IRS 0–4) and high (moderate-to-strong) (C-IRS 6–12) p300 expression (Table 2). Correspondingly, the percentage of cases with high cytoplasmic p300 expression increased significantly from nevi to melanoma (Figure 2D, E).

In order to explore, if the changes in p300 expression could be demonstrated in the same patient, we searched our database for matching cases in primary and metastatic melanoma stages. Our database included six cases which had samples matched for primary and metastatic melanoma. But two out of six cases already had low nuclear and high cytoplasmic expression at primary melanoma stage, which did not change any further in metastatic melanoma stage. The remaining four cases had high p300 expression in nucleus and cytoplasm at primary melanoma stage, which also did not change any further in metastatic melanoma stage.

Next, we studied the correlation between nuclear and cytoplasmic p300 expression using Spearman’s correlation test and χ^2^ test, to verify if there was any inverse correlation; an indicator of nuclear-to-cytoplasmic shift of p300 localization. As seen in Figure S5, there was a modest and positive correlation between nuclear and cytoplasmic scores. We found that though there were several cases which showed an inverse correlation, these results were overwhelmed by cases with similar levels of expression in nucleus and cytoplasm (Figure S5A).

### p300 Expression and Clinicopathologic Characteristics

Loss of nuclear p300 expression and increase in cytoplasmic p300 expression significantly correlated with the progression of melanoma from primary (AJCC stages I & II) to metastatic stage (AJCC III & IV) (Table 2). Further analysis revealed that cytoplasmic p300, but not nuclear p300 correlated positively with the tumor thickness (Table 2). To verify if the correlation between p300 expression and tumor thickness was more apparent in the initial stages, we analyzed the cases in the pT1 and pT2 stages [19]. However, we did not find any significant difference in p300 expression between pT1 and pT2 stages (data not shown).

UV radiation is a well known risk factor for melanoma [20] and p300 is known to play a role in the repair of DNA upon damage due to UV radiation [21], hence we studied the p300 expression in patients categorised as, tumor/s at sun-exposed sites and tumor/s at sun-protected sites. We found that patients with tumors on the sun-exposed sites tended to have lower p300 expression in the nucleus and higher p300 expression in the cytoplasm. But, the difference was not statistically significant (Table 2). We then performed the analysis in the patients with age greater than 60 years to explore the possible correlation between p300 expression and sun exposure in ageing patients. Although the tendency of correlation between low p300 expression in the nucleus and sun exposure status got stronger in the patients older than 60 years, the difference was still not statistically significant (Table 2).

We further continued with the analysis but we did not find any correlation between p300 expression and the rest of the demographic and clinicopathologic characteristics (Table 2).

### Loss of Nuclear p300 is Associated with Worse Patient Survival

**Figure 3:**
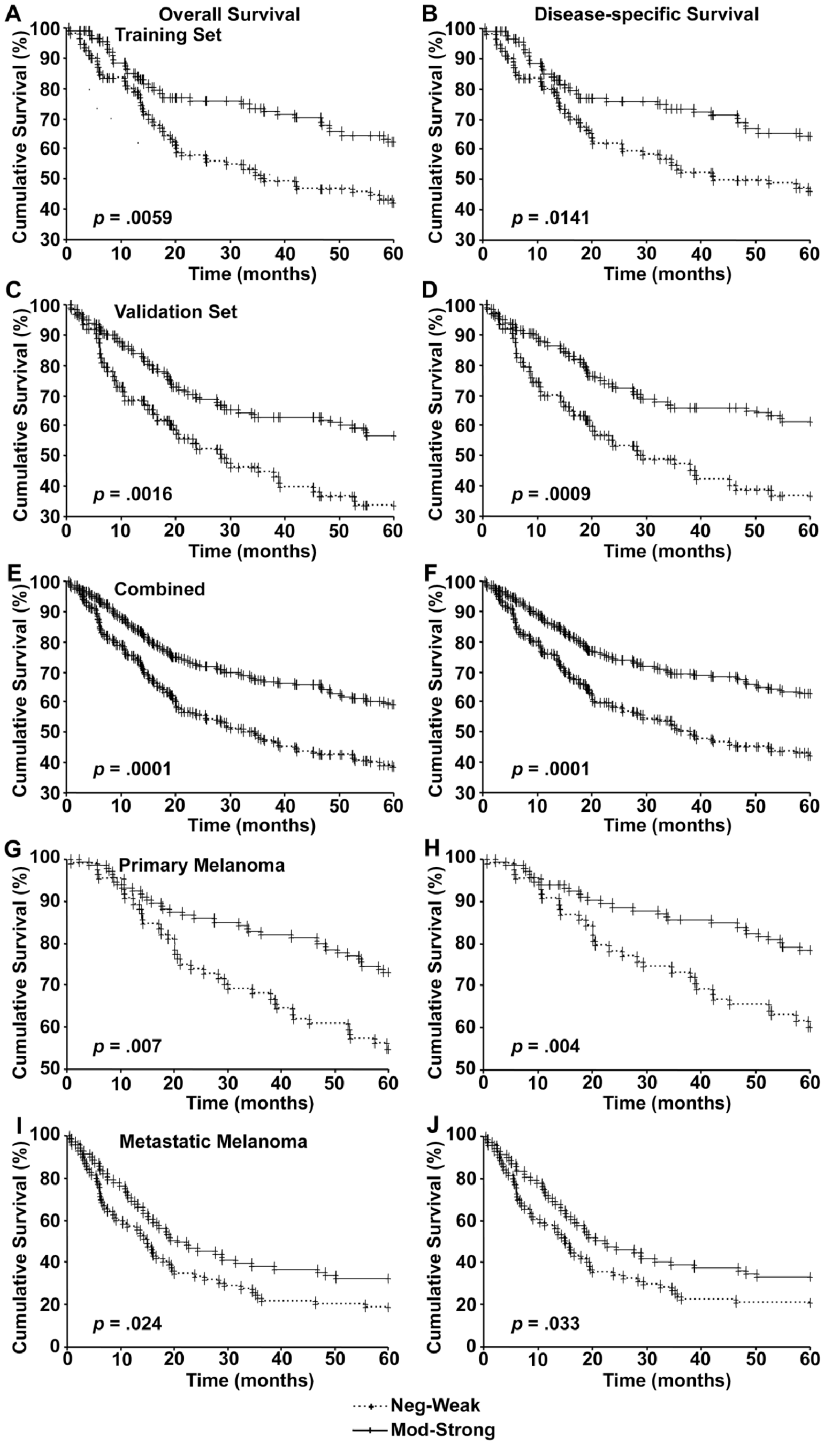
Nuclear p300 expression and 5-year patient survival. Kaplan-Meier survival analyses of correlation between nuclear p300 expression and 5-year overall (left panels) and disease-specific (right panels) survival of melanoma patients in the training set (A and B), validation set (C and D), two sets combined (E and F), and in primary (G and H) and metastatic melanoma patients (I and J).

Kaplan-Meier analysis of the survival of patients in the training set revealed that the patients with low nuclear p300 expression had significantly worse overall and disease-specific 5-year survival compared to patients with high nuclear p300 expression (Figure 3A, B). This was further confirmed by the analysis of patients included in validation set (Figure 3C, D) and the correlation between nuclear p300 expression and patient survival was even more significant when both data sets were pooled (Figure 3E, F). The cohort was then divided into primary melanoma and metastatic melanoma cases and the patient survival was analyzed as described above. Reduced nuclear p300 expression correlated with poor overall and disease-specific survival in both primary (Figure 3G, H) and metastatic melanoma patients (Figure 3I, J).

**Figure 4:**
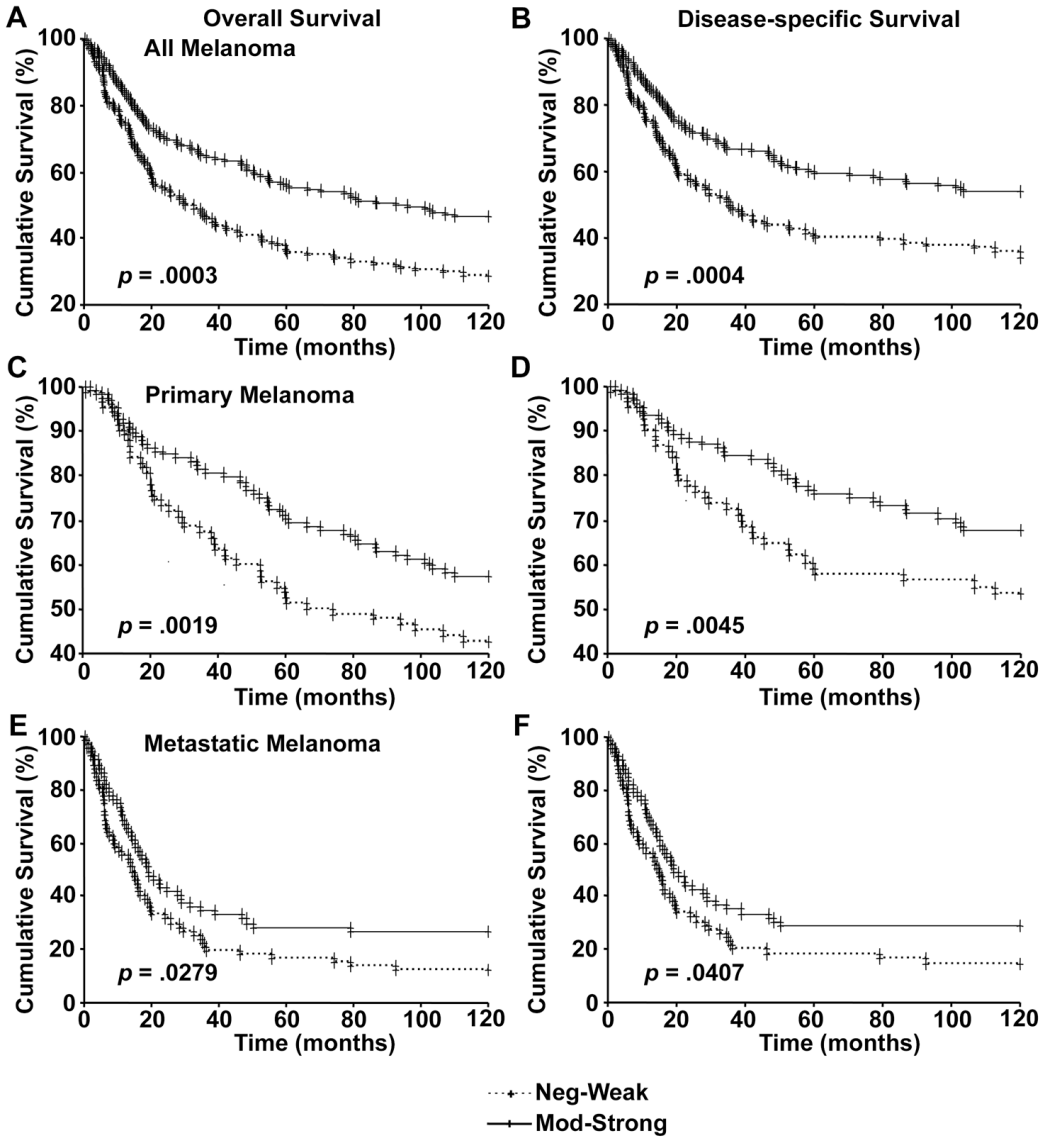
Nulcear p300 expression and 10-year patient survival. Kaplan-Meier survival analyses of correlation between nuclear p300 expression and 10-year overall (left panels) and disease-specific (right panels) survival of melanoma patients in all melanoma (A and B), primary melanoma (C and D), and metastatic melanoma (E and F).

Next we analyzed the data from patients where information regarding 10-year survival was available. As seen with 5-year survival, loss of nuclear p300 was associated with poor overall and disease-specific 10-year survival in all melanoma (Figure 4A, B), primary melanoma (Figure 4C, D), as well as metastatic melanoma patients (Figure 4E, F).

**Figure 5:**
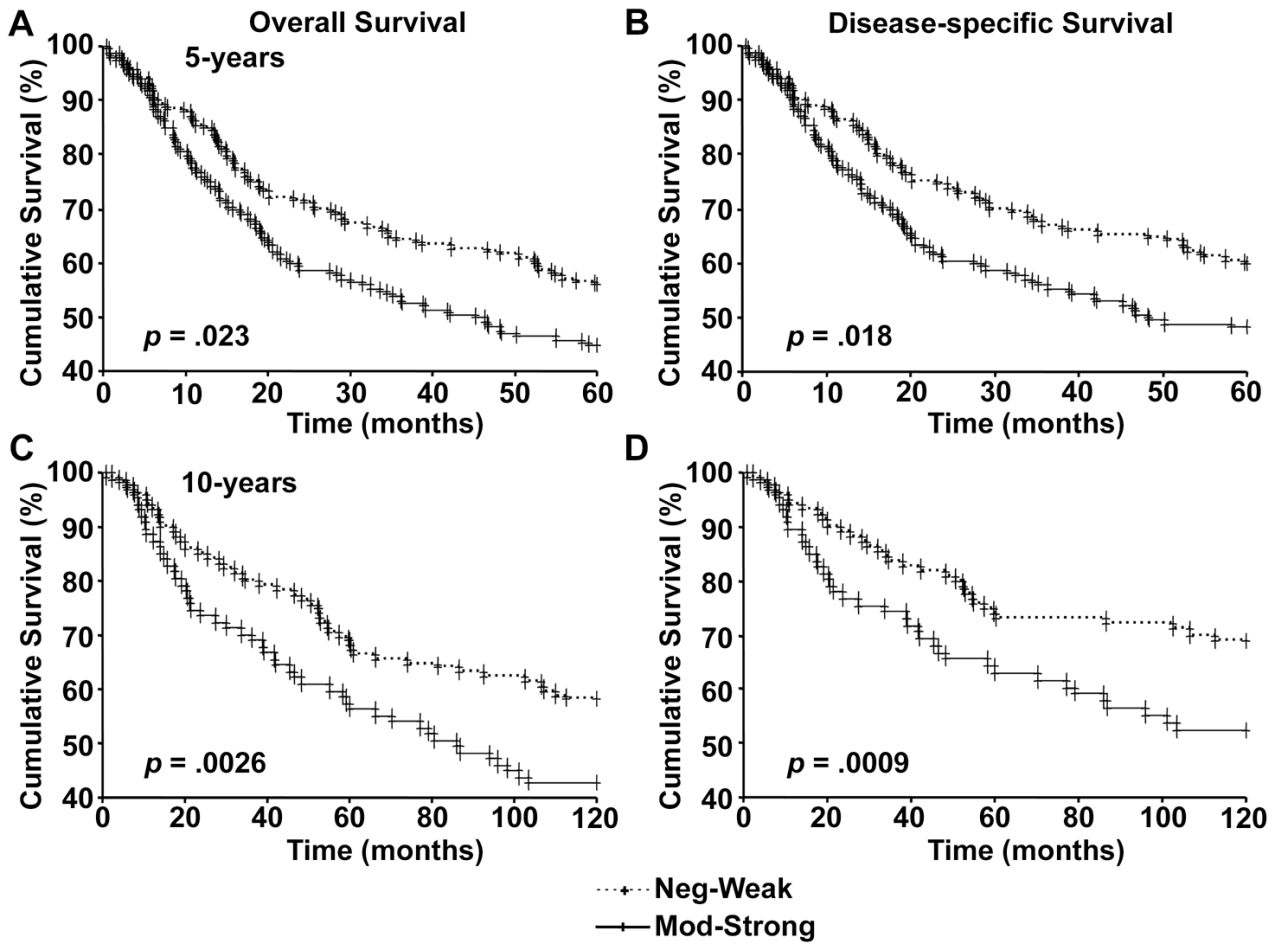
Cytoplasmic p300 expression and patient survival. Kaplan-Meier survival analyses of correlation between cytoplasmic p300 expression, and overall (left panels) and disease-specific (right panels) 5-year (A and B) and 10-year (C and D) survival of melanoma patients.

We then studied the significance of cytoplasmic p300 expression in the melanoma progression and patient survival. Increased cytoplasmic p300 expression was associated with worse overall and disease-specific survival in melanoma patients (Figure 5A–D). In order to examine the independence of p300 expression in the prediction of patient survival, we performed multivariate Cox proportional hazard analysis using AJCC stage, age, gender, nuclear p300 and cytoplasmic p300 as covariates. As seen in Table 3, nuclear p300, but not cytoplasmic p300, expression was an independent prognostic marker for both 5-year and 10-year melanoma patient survival.

**Table 3:** Multivariate Cox regression analysis of overall and disease-specific survival in melanoma patients.

Variables	Overall Survival				Disease-specific Survival			
	ß†	SE	HR (95% CI)	*p*-value	ß	SE	HR (95% CI)	*p*-value
**Training Set (5-year)**								
Nuclear p300	−0.549	0.225	0.59 (0.37–0.90)	.015	−0.479	0.234	0.62 (0.39–0.98)	.040
Cytoplasmic p300	0.275	0.221	1.32 (0.85–2.03)	.215	0.201	0.232	1.22 (0.78–1.92)	.387
AJCC	1.339	0.228	3.82 (2.44–5.97)	4.3×10^−9^	1.526	0.242	4.60 (2.86–7.39)	2.8×10^−10^
Gender	−0.092	0.234	0.91 (0.58–1.44)	.693	−0.135	0.246	0.87 (0.54–1.41)	.582
Age	0.253	0.222	1.29 (0.83–1.99)	.254	0.085	0.233	1.09 (0.69–1.72)	.715
**Validation Set (5-year)**								
Nuclear p300	−0.552	0.210	0.58 (0.38–0.87)	.009	−0.606	0.222	0.54 (0.35–0.84)	.006
Cytoplasmic p300	0.048	0.226	1.05 (0.67–1.63)	.833	0.140	0.244	1.15 (0.71–1.85)	.567
AJCC	1.287	0.232	3.62 (2.30–5.71)	3.0×10^−8^	1.470	0.251	4.35 (2.66–7.12)	4.6×10^−9^
Gender	0.465	0.215	1.59 (1.04–2.42)	.030	0.546	0.227	1.73 (1.11–2.69)	.016
Age	0.356	0.214	1.43 (0.94–2.13)	.096	0.317	0.226	1.37 (0.88–2.14)	.160
**Combined (5-year)**								
Nuclear p300	−0.548	0.152	0.58 (0.43–0.78)	3.0×10^−4^	−0.548	0.160	0.59 (0.42–0.79)	6.0×10^−4^
Cytoplasmic p300	0.163	0.155	1.18 (0.87–1.59)	.292	0.163	0.163	1.18 (0.85–1.62)	.317
AJCC	1.269	0.159	3.56 (2.61–4.86)	1.4×10^−15^	1.442	0.170	4.23 (3.03–5.90)	2.5×10^−17^
Gender	0.219	0.154	1.24 (0.92–1.68)	.157	0.234	0.162	1.26 (0.92–1.74)	.150
Age	0.326	0.151	1.38 (1.03–1.86)	.031	0.233	0.158	1.26 (0.93–1.72)	.141
**Combined (10-year)**								
Nuclear p300	−0.478	0.141	0.62 (0.47–0.82)	7.0×10^−4^	−0.482	0.152	0.62 (0.46–0.83)	.001
Cytoplasmic p300	0.266	0.143	1.30 (0.98–1.73)	.063	0.291	0.156	1.34 (0.98–1.81)	.062
AJCC	1.074	0.146	2.93 (2.20–3.89)	1.6×10^−13^	1.293	0.160	3.64 (2.66–4.98)	5.7×10^−16^
Gender	0.081	0.145	1.08 (0.82–1.44)	.575	0.164	0.156	1.18 (0.87–1.60)	.291
Age	0.442	0.141	1.56 (1.18–2.05)	.002	0.285	0.151	1.33 (0.99–1.79)	.059

Coding of variables: Age was coded as 1 ((60 years), and 2 (>60 years). Gender was coded as 1 (male) and 2 (female). p300 expression was coded as 1 (neg-weak) and 2 (mod-strong). AJCC staging was coded as 1 (stages I & II), and 2 (stages III & IV).

† ß: regression coefficient.

Abbreviations: SE, standard error of ß; HR, hazard ratio; CI, confidence interval.

Since, p300 expression was found to be associated with disease progression as well as survival, we asked if the expression was associated with the disease-free survival as well. We calculated the disease-free survival from the date of birth and the date of melanoma diagnosis and studied its correlation with p300 expression (nuclear and cytoplasmic) using Kaplan-Meier analysis. We, however, could not find any correlation between p300 expression and disease-free survival (data not shown). We suspect that in most patients, date of melanoma diagnosis does not reflect the actual onset of the disease and this could be the possible reason for the lack of correlation.

## Discussion

Genomic instability is now recognized as one of the major causes of cancer and the mechanisms regulating the integrity of genome are being investigated more intensely [9]. Since UV radiation, a well-known genotoxic agent, is one of the leading causes of melanoma [20], it is prudent to study the pathways and genes that regulate the genomic integrity. Histone acetylation, which leads to chromatin relaxation, is considered one of the important events during the process of DNA repair [5]. p300 is one of the most common HATs reported to be involved in DNA repair [4], [22]. Our data clearly showed that loss of nuclear p300 was associated with progression of disease into primary melanoma and then into metastatic melanoma. Understandably, loss of p300 might have led to persistence of oncogenic lesions on DNA thus leading to cancer. A previous study in human neonatal melanocytes showed a decrease in cellular p300 and CBP levels as the cells underwent replicative senescence [23]. Even in our analysis, we found that there was a slight increase in the percentage of strong nuclear p300 expression in dysplastic nevi compared to normal nevi. The loss of nuclear p300 expression was seen in later stages of melanoma.

The role of p300 and histone acetylation in the regulation of cell cycle regulator p27 (also known as p27^kip1^) expression has been reported [24]. Interestingly, nuclear expression of p27 was reportedly decreased during the progression of melanoma from normal and dysplastic nevi to primary and metastatic melanoma [25]. Our results thus highlight the need to conduct further studies on the correlation between p300 and p27 in melanoma. Pharmacological inhibition of p300 in melanoma cells has been recently reported to inhibit cell cycle progression [26]. But, we think that pharmacological inhibition of a protein does not completely reflect the decrease in abundance of the protein and further studies using ectopic expression of p300 as well as silencing of endogenous p300 in melanoma cells are needed to clarify the effect of p300 on melanoma cell cycle progression.

Previously, mutations in the region regulating the histone acetyltransferase activity of p300 have been reported in small cell lung cancer and B-cell lymphoma patients, [9], [10] but these studies did not report any association between the mutations and the patient survival. Moreover, to our knowledge, the expression of p300 has never been studied in melanoma patients. Our study for the first time showed that nuclear p300 can predict the patient survival independent of AJCC stage, age and gender. Similar to our results, loss of expression of acetylated histones was reported to be an independent prognostic factor for pancreatic and breast cancer patients [6], [8]. However, our results are in contrast to the findings in oesophageal and hepatocellular cancer, where high p300 expression correlated with worse survival in patients [12], [13]. We believe histone acetyltransferases in general, and p300 in particular, have a broad range of activities in the cell and their role could be different in different cell types.

Another important finding of our study is that the cytoplasmic expression of p300 in melanoma was increased compared to nevi. Cytoplasmic localization of p300 has been reported and some of its functions such as E4 ubiquitin ligase activity, were reported to be exclusive to cytoplasm [27]. Intriguingly, cytoplasmic p300, but not nuclear p300 expression correlated significantly with tumor thickness and showed a clear trend of correlation with ulceration status. Apparently, expression of p300 in cytoplasm confers a more invasive phenotype to melanoma cells. Along these lines, increase in cytoplasmic p300 correlated with worse survival in patients. But, our results did not present a strong association between cytoplasmic p300 expression and patient survival and moreover, it could not independently predict the patient survival. Therefore, we believe nuclear p300 is the regulatory factor for melanoma progression and emphasize the prognostic significance of nuclear p300 expression.

Nevertheless, our findings do point to the importance of cytoplasmic expression of p300 and encourages for further investigation. p300 protein is reported to harbour several sites of phosphorylation and site specific phosphorylations were shown to have varying effects on p300 function and stability [28]. While phosphorylation at serine-89 by protein kinase C (PKC) and salt inducible kinase 2 was shown to inhibit the HAT activity, phosphorylation at serine-1834 by Akt and serine-2279, serine 2315, and serine 2366 was shown to enhance the HAT activity [28], [29], [30], [31], [32]. Similarly, while Akt mediated phosphorylation was shown to stabilize the protein, interaction with mitogen activated protein kinase (MAPK) resulted in degradation of p300 [33], [34]. Yet, none of the studies focused on the effect of phosphorylation on p300 localization. Strikingly, p300 cytoplasmic localization was seen in A375 cell line, which has been reported to carry a homozygous BRAFV600E mutation [35] as well as in MM-RU cell line, which is reportedly a BRAF wild type [36]. Though it seems like BRAFV600E mutation status had no correlation with p300 cytoplasmic expression, our findings hint at a possible phosphorylation mediated re-distribution and regulation of p300 activity inside the cell by MAPkinase signalling, which warrants further investigation.

To date there is only a limited success with chemotherapy of metastatic melanoma [37]. Since we found that p300 was an independent prognostic factor, we think it would be worthwhile to either directly or indirectly target p300 for melanoma treatment. Histone deacetylase (HDAC) inhibitors, which prevent the deacetylation of histones, and indirectly potentiate the effects of histone acetyltransferases (p300), are commercially available and are under investigation for cancer treatment [38]. Two HDAC inhibitors, vorinostat (Merck) and romidepsin (Gloucester Pharmaceuticals), have been approved by the US FDA for the treatment of cutaneous T-cell lymphoma, a rare form of non-Hodgkin’s lymphoma that affects the skin [38]. Strikingly, both vorinostat and romidepsin were reported to have inhibitory effects on melanoma growth [39], [40]. It could be speculated that the patients with low nuclear p300 might find some improvement with these HDAC inhibitors. Nonetheless, it should also be noted that p300 is a complex protein with a variety of functions other than histone acetylation [27] and caution must be taken before concluding the benefits of HDAC inhibitors. Moreover, even though our database is relatively large and we had information on most of the clinicopathologic characteristics of patients, due to the retrospective nature of the study we could not include all the recognized prognostic factors in our analysis [19]. We did not have information on mitotic count or Ki67 expression, which is recently recommended by AJCC to be included in the criterion for melanoma staging [19]. We could not study the relationship between p300 expression and mitotic rate. Therefore, more studies with more patient information are needed to conclusively determine the usefulness of nuclear p300 in the melanoma treatment. Nevertheless, our study underscores the importance of targeting p300 (histone acetyltransferases) and provides encouragement for further studies in this direction.

In summary, we have shown the prognostic significance of nuclear p300 expression and also identified the importance of subcellular localization of p300 in the patient survival. Taken together, our study identifies a potential biomarker and molecular target for melanoma treatment.

## Supporting Information

Figure S1 Consort diagram showing patient exclusion and inclusion.
(DOC)

Figure S2 p300 expression in melanoma cells.
(DOC)

Figure S3 Representative images of strong p300 staining.
(DOC)

Figure S4 Identification of best cut-off IRS value for p300 expression.
(DOC)

Figure S5 Correlation between nuclear and cytoplasmic p300 expression.
(DOC)

Table S1 Demographics and clinical characteristics of 392 melanoma patients.
(DOC)
