# Supplementary figures and images for: Decreased Expression of Nuclear p300 Is Associated with Disease Progression and Worse Prognosis of Melanoma Patients
Source: PLoS One. 2013 Sep 30;8(9):e75405. doi: 10.1371/journal.pone.0075405 (PMC3787094; doi:10.1371/journal.pone.0075405)

**Figure S1. Consort diagram showing patient exclusion and inclusion.**

**
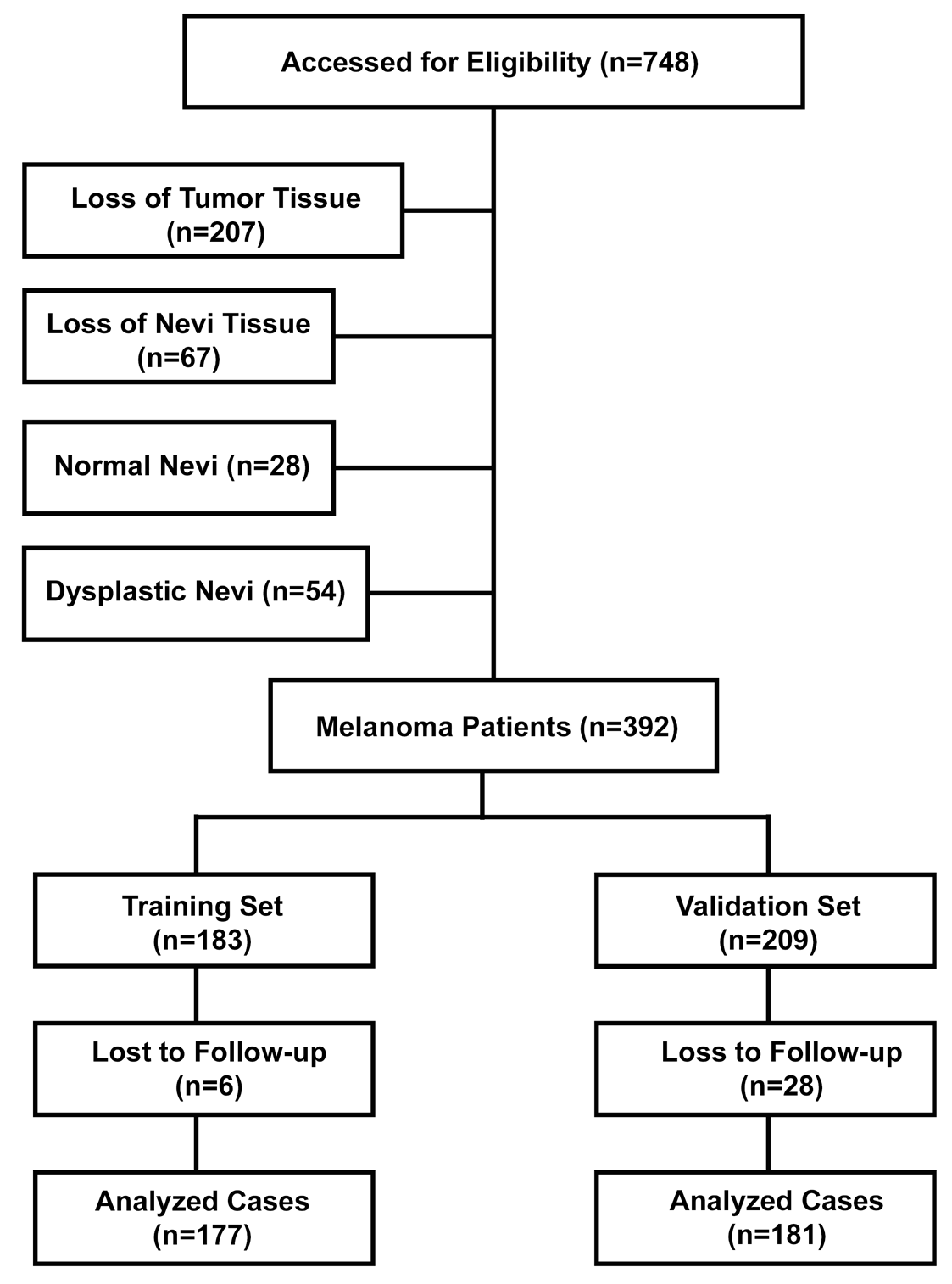
**

Supplement: Figure S1 — Consort diagram showing patient exclusion and inclusion. (DOC) [file pone.0075405.s001.doc]
