# Supplementary material for: Decreased Expression of Nuclear p300 Is Associated with Disease Progression and Worse Prognosis of Melanoma Patients
Source: PLoS One. 2013 Sep 30;8(9):e75405. doi: 10.1371/journal.pone.0075405 (PMC3787094; doi:10.1371/journal.pone.0075405)
Supplement: Figure S2 — p300 expression in melanoma cells. (DOC) [file pone.0075405.s002.doc]

**Figure S2. p300 expression in melanoma cells.** Immunofluorescent staining was performed on the cell lines using anti-p300 antibody (green). Nucleus was stained with DAPI (blue).

**
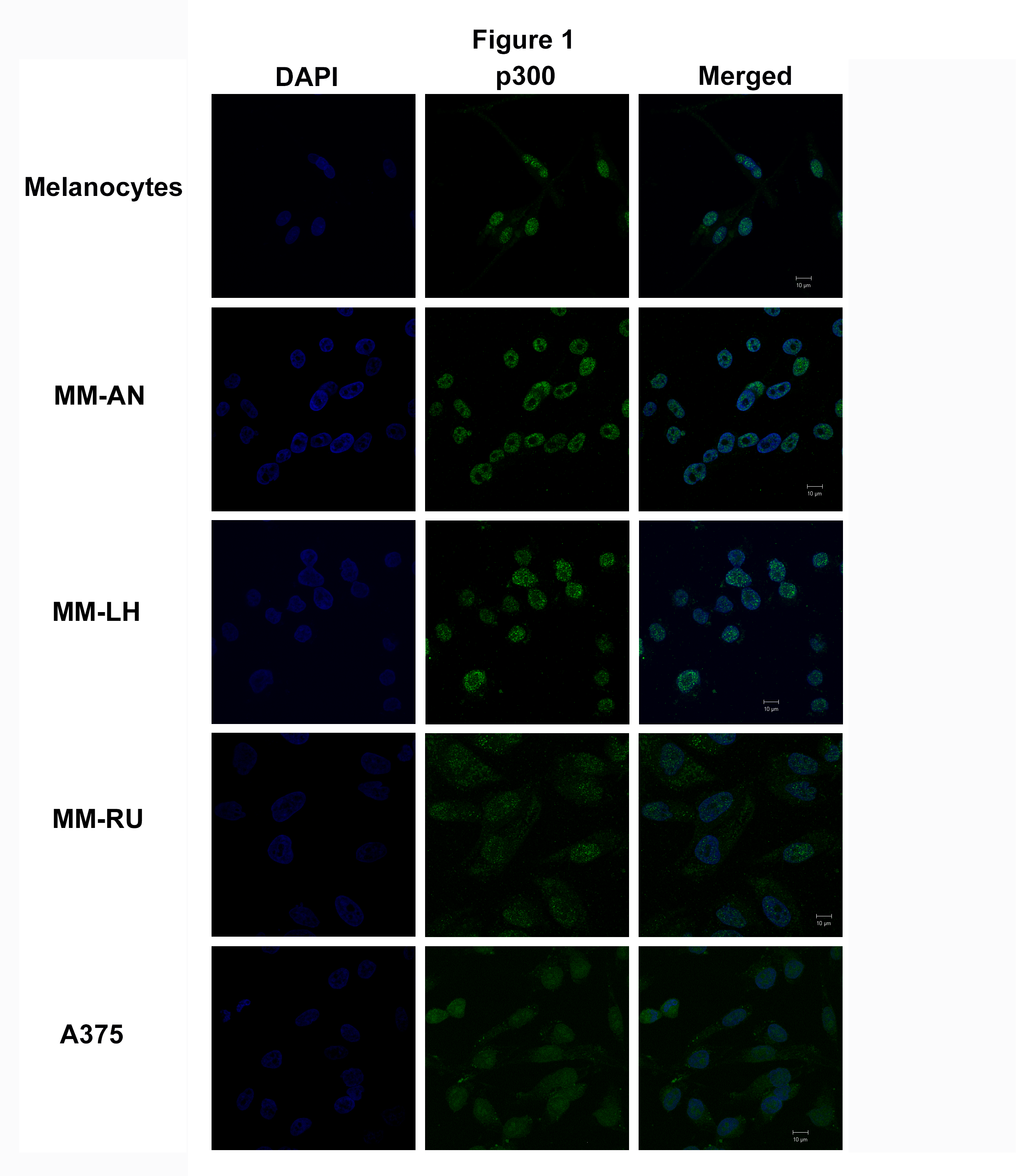
**
