# Supplementary material for: Decreased Expression of Nuclear p300 Is Associated with Disease Progression and Worse Prognosis of Melanoma Patients
Source: PLoS One. 2013 Sep 30;8(9):e75405. doi: 10.1371/journal.pone.0075405 (PMC3787094; doi:10.1371/journal.pone.0075405)
Supplement: Figure S3 — Representative images of strong p300 staining. (DOC) [file pone.0075405.s003.doc]

**Figure S3. Representative images of strong p300 staining.** Representative melanoma cores at 100× (upper panel) and 400× (lower panel) magnification of primary melanoma (PM) and metastatic melanoma (MM) for strong nuclear and cytoplasmic p300 expression.

**
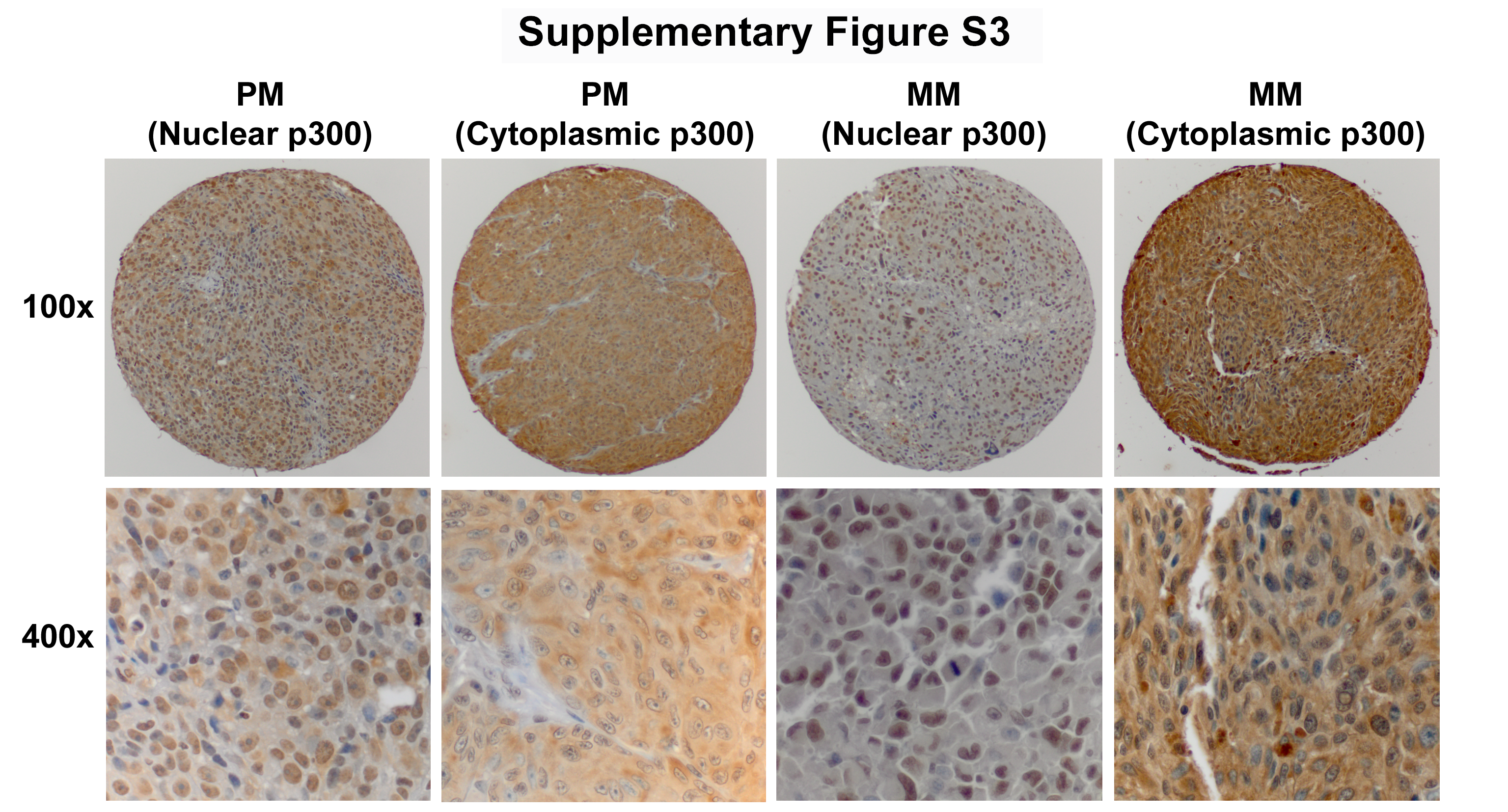
**
