# Supplementary material for: Decreased Expression of Nuclear p300 Is Associated with Disease Progression and Worse Prognosis of Melanoma Patients
Source: PLoS One. 2013 Sep 30;8(9):e75405. doi: 10.1371/journal.pone.0075405 (PMC3787094; doi:10.1371/journal.pone.0075405)
Supplement: Figure S4 — Identification of best cut-off IRS value for p300 expression. (DOC) [file pone.0075405.s004.doc]

**Figure S4. Identification of best cut-off IRS value for p300 expression. (**A) Nuclear p300 expression in normal nevi (NN), dysplastic nevi (DN), primary melanoma (PM), metastatic melanoma (MM). (B) Cytoplasmic p300 expression in normal nevi (NN), dysplastic nevi (DN), primary melanoma (PM), metastatic melanoma (MM).

**
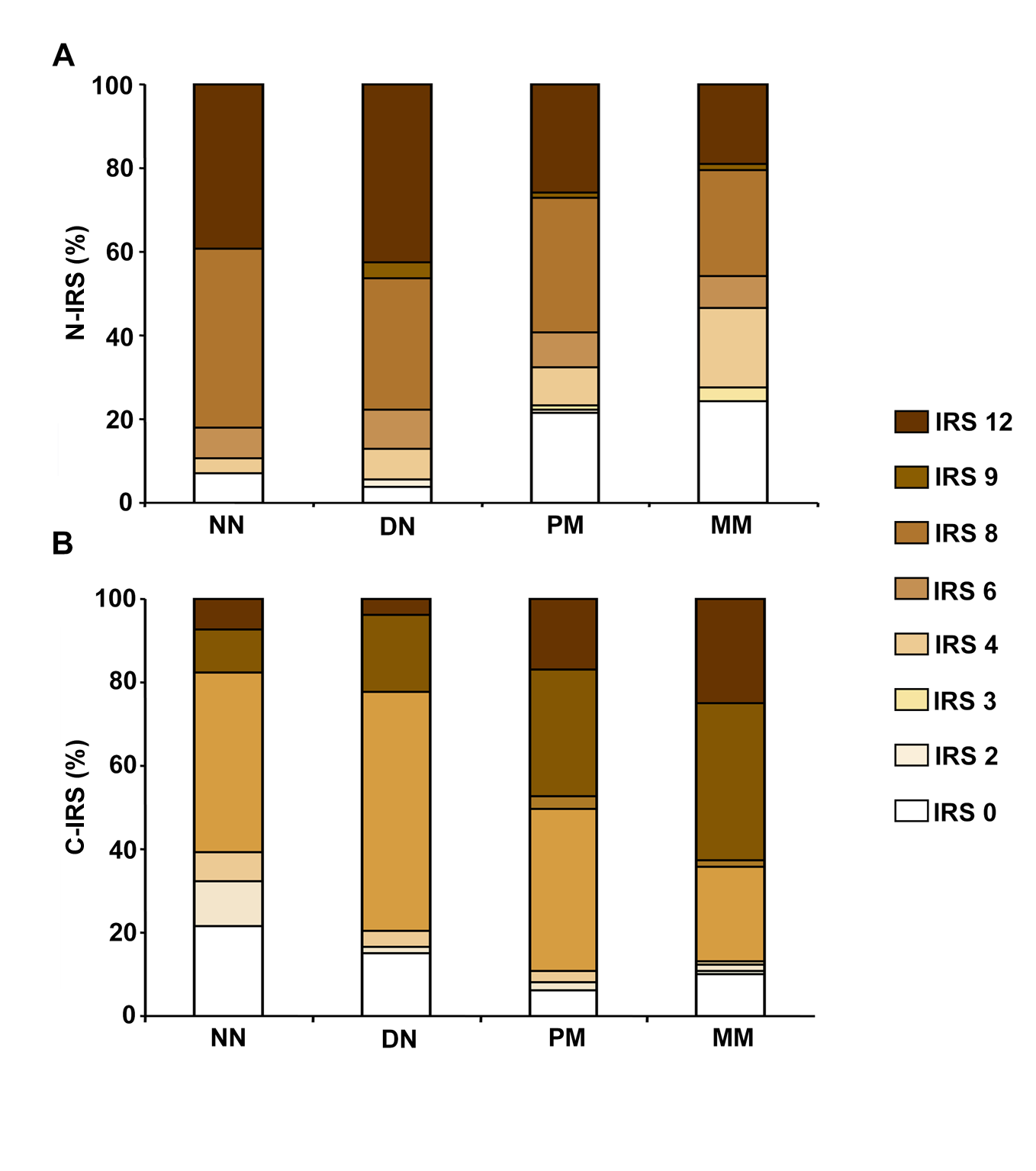
**
