# Supplementary material for: Decreased Expression of Nuclear p300 Is Associated with Disease Progression and Worse Prognosis of Melanoma Patients
Source: PLoS One. 2013 Sep 30;8(9):e75405. doi: 10.1371/journal.pone.0075405 (PMC3787094; doi:10.1371/journal.pone.0075405)
Supplement: Figure S5 — Correlation between nuclear and cytoplasmic p300 expression. (DOC) [file pone.0075405.s005.doc]

**Figure S5. Correlation between nuclear and cytoplasmic p300 expression (**A) Dot plot showing the distribution of nucleus (N-IRS) and cytoplasmic (C-IRS) scores across the tissue samples. p=0.0003 (Spearman’s correlation test, r=0.189) (B) Cytoplasmic p300 correlates positively with nuclear p300. p=0.03 (χ^2^ test).

**
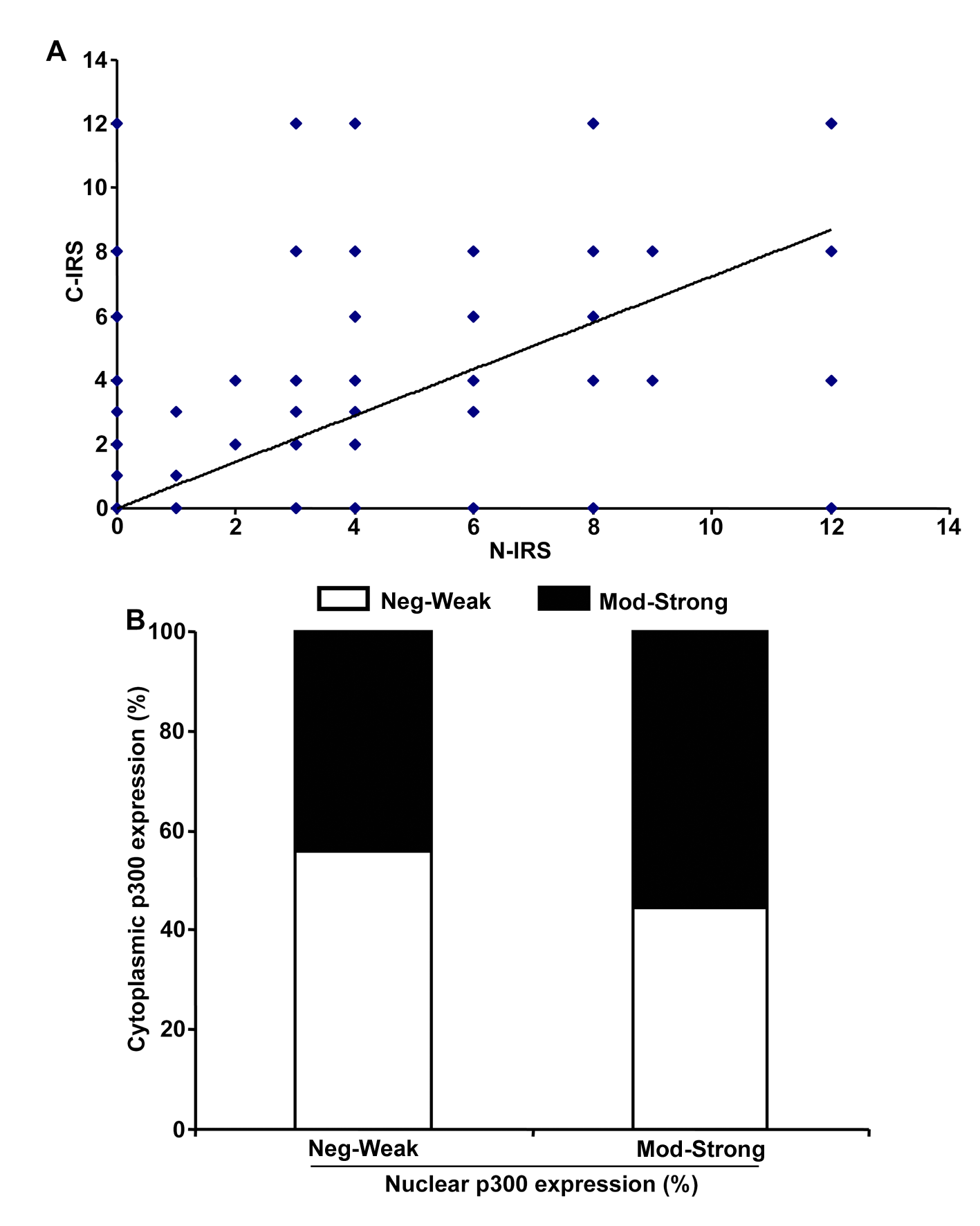
**
