# Supplementary material for: Decreased Expression of Nuclear p300 Is Associated with Disease Progression and Worse Prognosis of Melanoma Patients
Source: PLoS One. 2013 Sep 30;8(9):e75405. doi: 10.1371/journal.pone.0075405 (PMC3787094; doi:10.1371/journal.pone.0075405)
Supplement: Table S1 — Demographics and clinical characteristics of 392 melanoma patients. (DOC) [file pone.0075405.s006.doc]

**Table S1.** Demographics and clinical characteristics of 392 melanoma patients

| **Variables** | **Training Set** | **Validation Set** | **Total** | ***P*-value** |
| --- | --- | --- | --- | --- |
| **All Melanoma** (n=392) | | | | |
| Age  ≤ 60 | 102 (55.7%) | 98 (46.9%) | 200 (51.0%) |  |
| > 60 | 81 (44.3%) | 111 (53.1%) | 192 (49.0%) | .217 |
| Gender  Male | 118 (64.5%) | 113 (54.1%) | 231 (58.9%) |  |
| Female | 65 (35.5%) | 96 (45.9%) | 161 (41.1%) | .112 |
| AJCC  I | 60 (32.6%) | 65 (31.1%) | 125 (31.9%) |  |
| II | 59 (32.1%) | 67 (32.1%) | 126 (32.1%) |  |
| III | 22 (12.0%) | 37 (17.7%) | 59 (15.1%) |  |
| IV | 42 (22.8%) | 40 (19.1%) | 82 (20.9%) | .826 |
| **Primary Melanoma** (n=251) | | | | |
| Age  ≤ 60 | 69 (58.0%) | 52 (39.4%) | 121 (48.2%) |  |
| > 60 | 50 (42.0%) | 80 (60.6%) | 130 (51.8%) | .013 |
| Gender  Male | 74 (62.2%) | 64 (48.5%) | 138 (55.0%) |  |
| Female | 45 (37.8%) | 68 (51.5%) | 113 (45.0%) | .093 |
| Thickness  ≤ 2.0 mm | 67 (56.3%) | 76 (57.6%) | 143 (57.0%) |  |
| > 2.0 mm | 52 (43.7%) | 56 (42.4%) | 108 (43.0%) | .979 |
| Ulceration  Absent | 96 (80.7%) | 102 (77.3%) | 198 (78.9%) |  |
| Present | 23 (19.3%) | 30 (22.7%) | 53 (21.1%) | .805 |
| Site  Sun Protected | 93 (78.2%) | 92 (69.7%) | 185 (73.7%) |  |
| Sun Exposed | 26 (21.8%) | 40 (30.3%) | 66 (26.3%) | .3 |
| Subtype  Acrolentigous | 4 (3.4%) | 4 (3.0%) | 8 (3.2%) |  |
| Lentigous | 25 (21.0%) | 18 (13.6%) | 43 (17.1%) |  |
| Nodular | 19 (16.0%) | 26 (19.7%) | 45 (17.9%) | .681 |
| Spindle Type | 4 (3.4%) | 7 (5.3%) | 11 (4.4%) |  |
| Superficially Spreading | 44 (37.0%) | 43 (32.6%) | 117 (46.6%) |  |
| Unspecified | 23 (19.3%) | 34 (25.8%) | 57 (22.7%) |  |
| **Metastatic Melanoma** (n=141) | | | | |
| Age  ≤ 60 | 33 (51.6%) | 46 (59.7%) | 79 (56.0%) |  |
| > 60 | 31 (48.4%) | 31 (40.3%) | 62 (44.0%) | .622 |
| Gender  Male | 20 (31.3%) | 49 (63.6%) | 69 (48.9%) |  |
| Female | 44 (68.8%) | 28 (36.4%) | 72 (51.1%) | 6.5x10^-4^ |
